# Digenic Mutations in Junctional Epidermolysis Bullosa in An Iranian Family

**DOI:** 10.22074/cellj.2021.7208

**Published:** 2021-10-30

**Authors:** Kourosh Riahi, Farideh Ghanbari Mardasi, Farah Talebi, Farzad Jasemi, Javad Mohammadi Asl

**Affiliations:** 1. Department of Pediatrics, Faculty of Medicine, Ahvaz Jundishapur University of Medical Sciences, Ahvaz, Iran; 2. Department of Medical Genetics, School of Medicine, Tehran University of Medical Sciences, Tehran, Iran; 3. Department of Genetic, Faculty of Science, Shahid Chamran University of Ahvaz, Ahvaz, Iran; 4. Department of Internal Medicine, Faculty of Medicine, Ahvaz Jundishapur University of Medical Sciences, Ahvaz, Iran; 5. Department of Medical Genetics, Faculty of Medicine, Ahvaz Jundishapur University of Medical Sciences, Ahvaz, Iran

**Keywords:** *ITGB4*, Junctional Epidermolysis Bullosa Herlitz, *LAMA3*, *LAMB3*, Sequence Analysis

## Abstract

In this study, we describe one Iranian patient who was diagnosed with Epidermolysis Bullosa (EB) because of mutations in three candidate genes, including 3 mutations. Two missense mutations in the *LAMA3* (D3134H) and *LAMB3* (Y339H) genes and also, a synonymous mutation in the *ITGB4* (H422H) gene were identified that leads to the Junctional-EB- Herlitz (JEB-Herlitz) clinical phenotype. The patient had a heterozygous *LAMA3* mutation combined with a heterozygous mutation in *LAMB3*. Our results propose that these mutations produce novel protein-coding transcripts which explain the JEB-Herlitz phenotype in the patient. Interestingly, this is the first report indicating that a digenic inheritance in the *LAMA3* and *LAMB3* which is responsible for JEB-Herlitz. Also, this is the first digenic inheritance recognized in the JEB-Herlitz family. This study provides a new way to clarify the molecular mechanisms of *LAMA3* and *LAMB3* genes in JEB-Herlitz.

## Introduction

Epidermolysis Bullosa (EB) is the name used to define a heterogeneous group of inherited mechanobullous disorders that has been subdivided into three categories [EB simplex (EBS), dystrophic EB (DEB) and junctional EB (JEB)] based on the ultrastructural level of skin cleavage and immunofluorescence detection of cutaneous antigens (1-3). There are two major JEB subtypes, *JEB-Herlitz* (generalized), and JEB-non-Herlitz (localized) and each is typified by blister formation within the lamina lucida. JEB Herlitz is an autosomal recessive and severe form of EB that leads to the premature demise of the affected patients within a few months after birth. Many mutations in one of the 3 genes *LAMA3, LAMB3*, and *LAMC2* encode the a3, b3, and g2 subunit polypeptides of laminin 5 underlie this disease (4). In the present study, we performed next-generation sequencing (NGS) to identify the genetic mutations leading to JEB-Herlitz in an Iranian pedigree. 

### Case report

**Fig.1:**
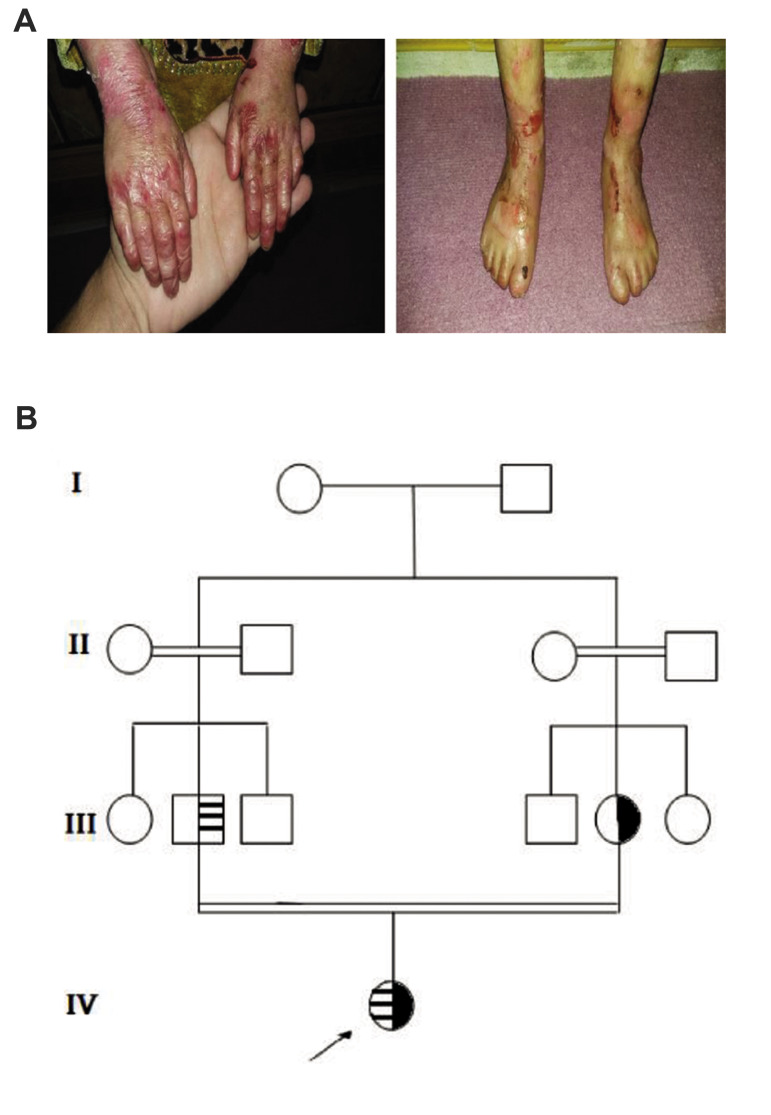
Clinical features and pedigree. **A.** Severe and widespread blistering in patient IV-1. **B.** Autosomal recessive inheritance pedigree. Areas with black color indicate maternal *LAMA3* mutations. 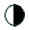; Horizontal stripes and 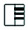; Paternal *LAMB3* mutations.

A 7-year-old Iranian girl, first child of consanguineous Iranian parents, was presented to our genetic counseling center because of widespread congenital skin blistering (JEB-Helitz) (Fig .1A). She had generalized blisters and erosions on her whole body, some dystrophic fingernails and toenails, with subungual hyperkeratosis and thickening of the nail plate. Hair involvement was limited to eyebrow alopecia. She did not have oral lesions. Also, in her unaffected parent, there was no previous family history of genetic diseases (Fig .1B).

After obtaining informed consent, genomic DNA was extracted from peripheral leukocytes of the patient, her parent, and 200 healthy controls by using the standard saltingout method (5). The study was performed in accordance with the Declaration of Helsinki and based on the guidelines of the Ethics Committee of Iran’s Ministry of Health and Medical. Sequence analysis was carried out by using a custom-designed (user-defined) NimbleGen chip capturing of 9 EB related genes followed by Next Generation Sequencing (NGS, BGI-Clinical Laboratories, Shenzhen, China). After NGS sequencing, the sequence reads were mapped to the reference human genomic DNA (UCSC/hg19) using the Burrows-Wheeler Alignment software (BWA v.0.7.10). Then, the subsequent variant was called with theGenome Analysis Toolkit (GATK) software versions 4 (https://software.broadinstitute.org/gatk/, GATK‐3.5) (6) to assemble the consensus sequence and detect single nucleotide polymorphisms (SNPs) and indels in target regions. Moreover, detected rare variants [minor allele frequency (MAF), 1%] in the affected girl were compared with database of SNP (dbSNP) (7) and 1000 genomes databases (8). Predicting candidate variants effect on protein structure and phylogenetic conservation, bioinformatics tools like PolyPhen-2 (9), SIFT (10) were used. And, the variant pathogenicity risk was estimated by CADD score (11). 

Then, direct Sanger sequencing was carried out with ABI3130 sequencer (Applied Biosystems, Foster City, CA, USA) to confirm potential causative variants in the patient. Primer sequences for pathogenic variants in the *LAMA3, LAMB3* and *ITGB4* genes (NM_198129, NM_000228 and NM_000213, respectively) were previously reported (12). Parent were examined for cosegregation analysis of the variants with the phenotype.

**Fig.2:**
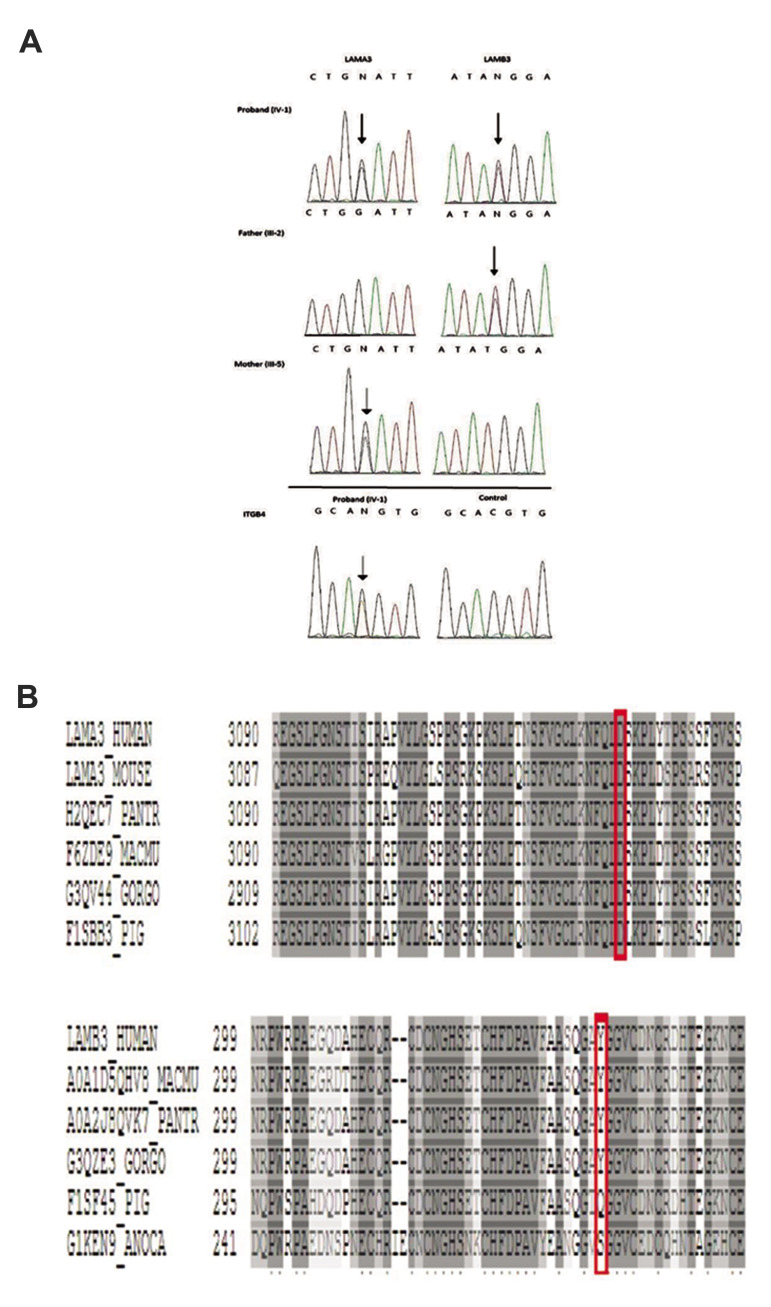
Digenic mutations in an Iranian family with Junctional Epidermolysis Bullosa (JEB). **A. **The result of DNA sequencing of the patient, father and mother. Patient: sequence analysis reveal a heterozygous G>C transversion at cDNA position 9641 of the *LAMA3* gene resulting in p.D3134H substitution, a heterozygous T>C transition at cDNA position 1405 of the *LAMB3* gene resulting in p.Y339H substitution and a heterozygous C>T transition at cDNA position 1266 of the *ITGB4* gene resulting in silent substitution p.H422H in the patient. Father: the father has p.Y339H substitution in the *LAMB3* gene. Mother: the mother has p.D3134H substitution in the *LAMA3* gene. (The p.D3134H, p.Y339H and p.H422H are marked with an arrow). **B. **Conservation analysis. Protein alignments show conservation of the amino acid sequence of *LAMA3* p.D3134H variant and *LAMB3* p.Y339H variant between species around the mutation sites that marked with vertical line (red).

Targeted exon capturing and NGS of 9 known EB related genes was performed in our patient. Among these genes, we detected 3 variants in the *LAMA3, LAMB3* and *ITGB4* genes in the patient which was absent in 200 healthy controls. Also, these variants were not previously reported in the same Iranian patients. Direct sequencing of the *LAMA3, LAMB3*, and *ITGB4* genes confirmed that the patient and her mother were heterozygous for c.9641 G>A mutation in exon 71 of the *LAMA3* gene (Fig .2A). This mutation (p. D3134H) affected a highly conserved amino acid residue (Fig .2B). Moreover, the patient and her father were found to carry a heterozygous c.1405 T > C in exon 9 of the *LAMB3* gene (p.Y339H) (Fig .2A, B). The patient also carried the c.1430 C>T mutation in a heterozygous state in the *ITGB4* gene (p.H422H) (Fig .2A). 

The segregate analysis confirmed these pathogenic mutations co-segregates with the disease phenotype in the patient. The family exhibited a typical autosomal recessive inheritance pattern of JEB-Herlitz (Fig .1B). Bioinformatics analysis was done by PolyPhen, SIFT and CADD (Table 1) and indicated that the p. D3134H and p. Y339H mutations together probably cause *LAMA3* and *LAMB3* dysfunction leading to the JEB-Herlitzclinical phenotype.

**Table 1:** *Various* in silico bioinformatics tools have been developed that predict the mutations


Gene	Prediction software		
	SIFT score	PolyPhen score	CAAD score

*LAMA3*	0.00 (Deleterious)	0. 9 (Probably damaging)	23 (Likely benign)
*LAMB3*	0.8 (Tolerated)	0.0 (Benign)	12 (Likely benign)


## Discussion

In this study, NGS was applied to identify the causative genes defects associated with EB in an Iranian pedigree. The index patient was a double-heterozygous carrier for two missense mutations in the *LAMA3* and *LAMB3* genes. So far, researchers reported eighteen missense mutations in the *LAMA3* gene based on the HGMD database (13). Our first identified mutation, (p. D3134H), in the patient and her mother, was in the laminin G-like 4 (LG-4) domain of *LAMA3* protein C-terminal that leads to loss negatively charged side chains and replaced by a positively charged residue. The second identified mutation in the proband and her father, c.1405T>C, was a heterozygous mutation in the laminin epidermal growth factor-like 2 (EGF-like 2) domain of the LAMB3 protein. Although, these mutations have previously been reported, this is the first report of mutations of *LAMA3* and *LAMB3* genes in an Iranian EB patient. Following evidences prove that these mutations can lead to EB: i. Next generation sequencing only identified these mutations to be the main cause of EB in the patient. ii. Direct Sanger sequencing proved the mutations in the proband and also, based on recognizing heterozygote mutations in her parents, the pattern of inheritance must be an autosomal recessive and digenic. iii. Using predicting online tools such as SIFT, polyphen, CADD , these variants will be damaging and tolerated (p.D3134H and p.Y339H, respectively). iv. The amino acids comparative alignment of LAMA3 and LAMB3 proteins across all Kingdoms showed that p. D3134 of *LAMA3* gene is highly conserved during evolution. v. Also, a substitution Asp3134His in *LAMA3* gene and a substitution Tyr339His in *LAMB3* gene can create major problems in the LAMA3 and LAMB3 proteins. Thus, these mutations in *LAMA3* and *LAMB3* genes are pathogenic in our patient with EB.

According to simplified Schäffer definition, the most part of cases in digenic diseases are categorized into two classes (14). The first class represents true digenic (TD) instances: variants at both loci are essential for disease and, variants at one of the two loci lead to no phenotype (15). The second class we will refer to as the composite (CO) class as it consists of diverse possibilities: A composite case in digenic diseases can refer to mendelizing variants plus modifiers, when a driver variant is essential for the phenotype but rare variants in a second gene, generally correlated to the same pathway, may change the phenotype (16).

All involved variants impact, the genes allelic condition, the gene ability of enduring loss of function (LOF) variants, and also, the involved genes correlation are likely to identify the digenic effect. Several common properties of digenic combinations are characteristic for the two classes, and somehow reflect the underlying biological mechanisms. The digenic effect is often strongly influenced by the impact of the variants implicated as well as their zygosity (17).

The digenic inheritance in genes has been reported in some human phenotypes, for example, retinitis pigmentosa (18, 19), non-syndromic hereditary deafness, Wardenburg syndrome type 2, Bardet-Biedl syndrome, autosomal recessive ocular albinism, JEB and EBS (20, 21). Previously, digenic inheritance has been described in a case with severe nonlethal JEB (JEB- non-Herlitz), in which one mutation in the *LAMB3* gene and two mutations in the type XVII collagen gene were identified (22). The collagen XVII and Laminin-5, two functionally related proteins, abnormal expression led to the primary hemidesmosome structure and the basement membrane separation of the epidermis, with severe skin blistering as the clinical appearance. Also, digenic inheritance was reported in three previous cases with EBS in which mutations occur in *KRT5* and *KRT14* genes (Table 2) (23-25). 

The fact that the p.D3134H (in *LAMA3*) and p.T339H (in *LAMB3*) mutations reported in present study affects an extremely conserved residue, supports a positive pathogenic role for these genes in causing the disease phenotype. Therefore, these results propose that digenic inheritance was directly involved in modifying/causing the clinical phenotype in this patient.

As a rare disease, this is the first report that indicated a JEB-Herlitz responsible digenic inheritance of *LAMA3* and *LAMB3*. Also, this is the first digenic inheritance recognized in an Iranian JEB-Herlitz family. 

**Table 2:** Previous studies on the digenic inheritance in EB


Origin t	Type of EB	Genes	Pathogenic variant	Protein effect	Type of mutation	Method	

German	JEB	*COL17A1*	c. T2669Gc. C3781T	L855XR1226X	MissenseMissense	candidate gene sequencing	
		*LAMB3*	c. C1903T	R635X	Missense	candidate gene sequencing	
Jewish Ashkenazi	EBS	*KRT5*	c. T548C	p.I183T	Missense	candidate gene sequencing	
		*KRT14*	c. G1163A	p.R388H	Missense	candidate gene sequencing	
Australian	EBS	*KRT5*	c.464T>C	p. Leu155Pro	Missense	candidate gene sequencing	
		*KRT14*	c.881T>C	p. Met294Thr	Missense	candidate gene sequencing	
Polish	EBS	*KRT5*	c.1412G>A	p.Arg471His	Missense	candidate gene sequencing	
		*KRT14*	c.815T>C	p.Met272Thr	Missense	candidate gene sequencing	
Iranian	JEB-Herlitz	*LAMA3*	c. G9641C	p. D3134H	Missense	candidate gene sequencing	
		*LAMB3*	c. T1405C	p.Y339H	Missense	candidate gene sequencing	


EB; Epidermolysis Bullosa, JEB; Junctional-EB, and EBS; EB simplex.

## Conclusion

We emphasize that one mutation detection in one gene is not sufficient for determining the molecular basis of JEB-Herlitz in a given family. Moreover, we present evidence implicating digenic inheritance in identifying a clinical phenotype in JEB-Herlitz, proposing that full sequencing of all JEB-Herlitzrelated genes may develop the quality of genetic counseling and prenatal diagnosis of affected individuals in this clinically heterogeneous disease.
